# Effects of a novel cyclic RGD peptidomimetic on cell proliferation, migration and angiogenic activity in human endothelial cells

**DOI:** 10.1186/2045-824X-6-11

**Published:** 2014-05-21

**Authors:** Roberto Fanelli, Laura Schembri, Umberto Piarulli, Monica Pinoli, Emanuela Rasini, Mayra Paolillo, Marisa Carlotta Galiazzo, Marco Cosentino, Franca Marino

**Affiliations:** 1 Department of Science and High Technology, University of Insubria, Como, Italy; 2 Center for Research in Medical Pharmacology, University of Insubria, Via Ottorino Rossi n. 9 21100, Varese, VA, Italy; 3 Department of Drug Sciences, University of Pavia, Pavia, Italy

**Keywords:** RGD peptidomimetics, Integrins, Angiogenesis, Human umbilical vein endothelial cells, Interleukin-8

## Abstract

**Background:**

Cyclic RGD peptidomimetics containing a bifunctional diketopiperazine scaffold are a novel class of high-affinity ligands for the integrins α_V_β_3_ and α_V_β_5_. Since integrins are a promising target for the modulation of normal and pathological angiogenesis, the present study aimed at characterizing the ability of the RGD peptidomimetic *cyclo*[DKP-RGD] **1** proliferation, migration and network formation in human umbilical vein endothelial cells (HUVEC).

**Methods:**

Cell viability was assessed by flow cytometry and annexin V (ANX)/propidium iodide (PI) staining. Cell proliferation was evaluated by the ELISA measurement of bromodeoxyuridine (BrdU) incorporation. Network formation by HUVEC cultured in Matrigel-coated plates was evaluated by optical microscopy and image analysis. Integrin subunit mRNA expression was assessed by real time-PCR and Akt phosphorylation by western blot analysis.

**Results:**

*Cyclo*[DKP-RGD] **1** does not affect cell viability and proliferation either in resting conditions or in the presence of the pro-angiogenic growth factors VEGF, EGF, FGF, and IGF-I. Addition of *cyclo*[DKP-RGD] **1** however significantly decreased network formation induced by pro-angiogenic growth factors or by IL-8. *Cyclo*[DKP-RGD] **1** did not affect mRNA levels of α_V_, β_3_ or β_5_ integrin subunits, however it significantly reduced the phosphorylation of Akt.

**Conclusions:**

*Cyclo*[DKP-RGD] **1** can be a potential modulator of angiogenesis induced by different growth factors, possibly devoid of the adverse effects of cytotoxic RGD peptidomimetic analogues.

## Introduction

Angiogenesis, the growth of new blood vessels as sprouts or offshoots of the pre-existing microvasculature, is a physiological event occurring in the development of organisms, wound healing and the reproductive cycle, but it is also involved in pathologic processes such as inflammation, tumour growth and metastasis [1]. Angiogenesis can be stimulated by a large number of pro-angiogenic cytokines, such as vascular endothelial growth factor (VEGF), tumour necrosis factor α (TNF-α), basic fibroblast growth factor (bFGF) and interleukin-8 (IL-8) [2,3].

Among the proteins involved in the angiogenic process, integrins play an important role by promoting endothelial cell attachment and migration on the surrounding extracellular matrix, cell to cell interaction and intracellular signal transduction [4]. Integrins are heterodimeric proteins composed of two non covalently associated α and β transmembrane glycoproteins; 18 α and 8 β subunits that give rise to 24 possible distinct integrin proteins [5,6]. Across their extracellular α/β subunit interface containing the metal ion-dependent adhesion site (MIDAS), integrins recognize and bind protein ligands through contiguous tripeptide sequences, the majority of which are present within flexible loop regions and contain an acidic residue [7]. Several integrins, including α_V_, α_5_β_1_ and α_IIb_β_3_ integrins, recognize the Arg-Gly-Asp (RGD) sequence in endogenous ligands. The context of the ligand RGD sequence (flanking residues, three dimensional presentation) and individual features of the integrin binding pockets determine the recognition specificity and efficacy. These observations prompted many research groups to investigate the use of conformationally constrained cyclic RGD peptides and peptidomimetics as active and selective integrin ligands [8,9]. One of these, Cilengitide, namely cyclo-[Arg-Gly-Asp-D-Phe-N(Me)-Val] is currently in phase III clinical trials as an angiogenesis inhibitor for patients with glioblastoma multiforme alone [10] or in combination with other antiblastic drugs [11]. Recently, RGD compounds have been proposed also as targeting ligands for integrins in order to better characterize tumor neovascularisation [12]. Notwithstanding these results, the mechanism of RGD ligands in the inhibition of angiogenesis is not yet fully understood, as significant cross-talk exists in the regulation of angiogenesis between integrin operated pathways and, for instance, VEGF receptor pathways [13], and on these bases it has been proposed that agents able to inhibit multiple pathways would have important therapeutic potential [14].

**Figure 1:**
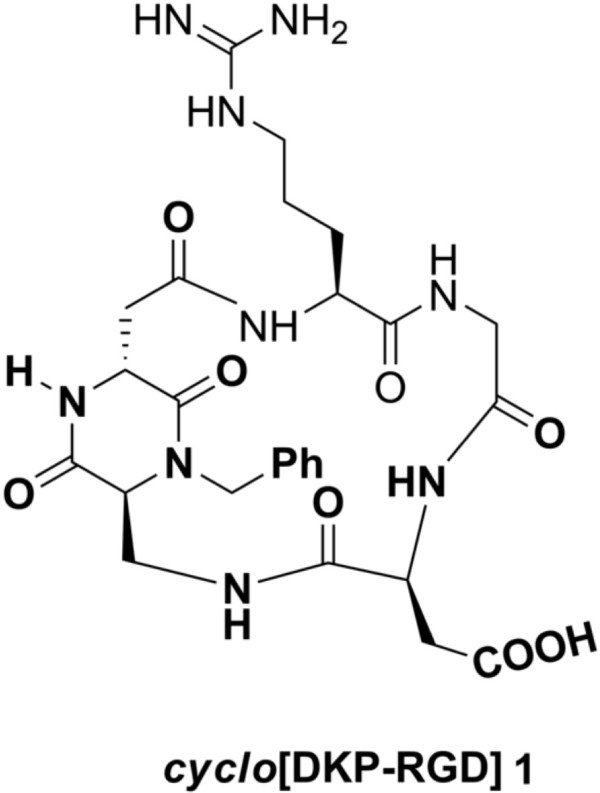
**Structure of the peptidomimetic *cyclo*** **[DKP-RGD] 1.**

Recently, some of us reported a new class of cyclic RGD peptidomimetics containing a bifunctional diketopiperazine (DKP) scaffold, showing a low nanomolar affinity for integrins α_V_β_3_ and α_V_β_5_[15,16]. The present study is aimed at characterizing the ability of the cyclic RGD peptidomimetic *cyclo*[DKP-RGD] **1** (Figure 1) to affect cell viability, proliferation, migration and capillary network formation in human umbilical vein endothelial cells (HUVEC). In addition, the effect of *cyclo*[DKP-RGD] **1** on mRNA expression of the integrin subunits α_V_, β_3_ and β_5_, and on the phosphorylation of Akt, a serine/threonine-specific protein kinase that plays a key role in the regulation of vascular homeostasis and angiogenesis [17] was also investigated.

## Materials and methods

### Reagents

The peptidomimetic *cyclo*[DKP-RGD] **1** was prepared according to a published procedure [16]. Annexin V-FITC Apoptosis Detection Kit I was purchased by Biosciences (BD, Italy). RNA extraction was performed using Quiazol reagent (Qiagen, Italy) and the quantitative real time RT-PCR reaction were performed using Quantitatec reverse transcription kit (Qiagen, Italy) and Quantitec sybr green pcr kit (Qiagen, Italy). The amount of proteins for western blot analysis were performed by the BCA protein Assay Kit (Pierce Protein Biology, Rockford, IL, USA). Anti-AKT and anti-pAKT, primary antibodies were purchased from Cell Signalling (Cell signalling, Italy) and horseradish peroxidase-conjugated goat anti-rabbit IgG secondary antibody for western blot were purchased from Upstate Biotechnology (Upstate Biotechnology, USA). The detection of the western blot membrane was performed by using ECL plus Western Blotting Detection System purchased from Amersham (Amersham, GE Healthcare Life Science, MI, Italy). Propidium iodide (PI) solution was purchased from Miltenyi (Miltenyi Biotec S.r.l., Bologna, Italy). Matrigel Basement Membrane Matrix (10 mg/ml) for the network formation assay was purchased from BD (Becton Dickinson Italy, Milan, Italy). Cell proliferation Biotrak Ver for the proliferation assay was purchased from GEhealthcare (GeHealthcare, Uppsala, Sweeden). Human umbilical vein endothelial cells (HUVEC) were obtained from PromoCell (PromoCell Gmbh, Germany). The EndoGRO™ VEGF Complete Media Kit composed of EndoGRO Basal Medium (SCME-BM) plus fetal bovine serum (FBS), L-glutamine, heparin sulphate, rh-VEGF, rh-EGF, rh-FGF2, rh-IGF-I and ascorbic acid, and the MF-Membrane filters (3.0 μm) for the cell migration assay were purchased from Millipore (Millipore S.p.A., MI, Italy). Recombinant Human CXCL8/IL-8 was purchased from R&D (R&D System, US, Europe).

### Cell cultures

HUVEC were cultured in a medium supplemented with FBS (2%), L-glutamine (10 mM), heparin sulphate (0.75 U/ml), VEGF (5 ng/ml), EGF (5 ng/ml), FGF2 (5 ng/ml), IGF-I (15 ng/ml) and ascorbic acid (50 μg/ml) at 37°C, in a moist atmosphere of 5% CO_2_. HUVEC were used for the experiments between passage 2 and 8. All the experiments were conducted under two different conditions: basal conditions (resting) i.e. cell cultured in EndoGRO basal medium alone, and stimulated conditions, i.e. with the addition of VEGF (5 ng/ml), EGF (5 ng/ml), FGF2 (5 ng/ml), IGF-I (15 ng/ml) together with 10% FBS. In viability, proliferation and migration assays, cells were used after overnight culture in EndoGRO basal medium alone (starvation).

### Cell viability

Cell viability assay was performed by flow cytometry. Briefly, after treatment HUVEC were detached with a trypsin solution, centrifuged at 600 g for 5 min at room temperature and the supernatant was finally removed. The cell pellet was resuspended in 100 μL Binding Buffer 1× with the addition of 5 μL annexin V (ANX)-FITC and 5 μL PI, and finally incubated for 15 min at room temperature in the dark. Samples were stored on ice and analyzed without washing. Acquisition was performed on a BD FACSCanto II flow cytometer (Becton Dickinson Italy, Milan, Italy) and data were analyzed using BD FACSDiva software (version 6.1.3). HUVEC were identified on the basis of forward-scatter (FSC) and side-scatter (SSC) properties, and a minimum of 15000 cells for each sample was collected in the gate. Viable, apoptotic and necrotic HUVEC were identified on a biparametric plot ANX-FITC vs PI. Data were finally expressed as% viable (ANX-/PI-), early apoptotic cells (ANX+/PI-), late apoptotic/necrotic cells (ANX+/PI+) and necrotic cells (ANX-/PI+).

### Proliferation assay

To assess HUVEC proliferation, 1×10^4^ cells were seeded in duplicate in a 96-well plate and cultured for 24 h without or with *cyclo*[DKP-RGD] **1** at different concentrations. Proliferation was then measured by a colorimetric immunoassay, based on the ELISA measurement of bromodeoxyuridine (BrdU) incorporation during DNA synthesis. The absorbance (ABS) of the samples was determined by means of a spectrophotometer (Model 680, Bio-Rad Laboratories, Hercules, CA, USA) with wavelength set at 450 nm, and finally expressed as the difference between BrdU positive and negative samples, expressed as Optical Density (O.D.).

### Cell migration assay

Cell migration was measured by means of a Boyden chamber assay. Briefly, 1×10^5^ HUVEC were seeded in the top well of the Boyden chamber, *cyclo*[DKP-RGD] **1** was added in the bottom or in the top compartment, and a 3 μm-pore cellulose nitrate filter was placed between the two compartments. Stimulated migration was assessed by putting VEGF, EGF, IGF-I, and FGF2 in the bottom chamber. After an incubation period of 5 h at 37°C, the filter was recovered, dehydrated, fixed, and finally stained with hematoxylin. Migration into the filter was quantified by measuring the distance (in μm) from the surface of the filter to the leading front of cells using an optical microscope (Axiolab, Carl Zeiss S.p.A. Milan, Italy).

### Angiogenesis assay

To assess angiogenic activity, HUVEC 2.5×10^4^ cells were seeded in a 24-well plate coated with 100 μl/well of Matrigel previously polymerized for 1 h at 37°C. Cells were then incubated for 5 h at 37°C in a moist atmosphere of 5% CO_2_ without or with *cyclo*[DKP-RGD] **1** under either resting or stimulated conditions. In some experiments IL-8 (10 nM) was used as pro-angiogenic stimulus. Network formation was evaluated by phase‒contrast microscopy using a fluorescence microscope (Axiovert 40CFL, Carl Zeiss S.p.A. Milan, Italy). Network formation was finally quantified in terms of mean number of loops per field as topological parameters and the total length of the branches. For the purpose of the analysis, loops were defined as any complete ring formed by HUVEC, while open ramifications were considered as branches. The total branch length (pixels) and the number of loops were quantified using the ImageJ image analysis software (http://rsbweb.nih.gov/ij/).

### Real time PCR

Cells were treated for 5 h in the presence or absence of 1 μM cyclo[DKP-RGD] 1 in different growth conditions, as previously described. At the end of the treatment, RNA extraction was performed using the Qiazol lysis reagent. Primers were designed by using the “Primer3 input” software (http://frodo.wi.mit.edu/cgi-bin/primer3/primer3.cgi/primer3_www.cgi) and the specificity of each primer was controlled by the BLAST software (http://blast.ncbi.nlm.nih.gov) (Table 1). Real time PCR was performed as previously reported [18] At the end of the reaction, a melting curve analysis was carried out to check for the presence of primer-dimers. Comparison of the expression of each gene was determined by using GAPDH as housekeeping gene. Each run was analyzed in duplicate and data are finally expressed as 2^-Δct^.

**Table 1:** Sequences of the primers and PCR products size

**Gene**	**Ref. sequence**	**Sequence**	**Product size**
α_v_	NM_002210	Forward: actggcttaagagagggctgtg	110
		Reverse: tgccttacaaaaatcgctga	
β_3_	NM_000212	Forward: agacactcccacttggcatc	123
		Reverse: tcctcaggaaaggtccaatg	
β_5_	NM_002213	Forward: agcctatctccacgcacact	91
		Reverse: cctcggagaaggaaacatca	
GAPDH	NM_001289746.1	Forward: caactgtgaggaggggagatt	97
		Reverse: cagcaagagcacaagaggaag	

### Western blot analysis

Cells grown in 60-mm dishes were treated for 5 h with 1 μM *cyclo*[DKP-RGD] 1. The cells were then rinsed twice in ice-cold PBS and 200 μl of the cell lysis buffer (composition: 50 mM Tris–HCl pH 7.4, 1% v/v NP40, 0.25% w/v sodium deoxycholate, 1 mM phenylmethylsulphonyl-fluoride, 1 mM Na3VO4, 1 mM EDTA, 30 mM sodium pyrophosphate, 1 mM NaF, 1 mg/ml leupeptin, 1 mg/ml pepstatin A, 1 mg/ml aprotinin and 1 mg/ml microcystin) was added to the dishes. After scraping, cells were sonicated for 10 s, centrifuged at 12000 g for 5 min at 4°C and the amount of proteins in the supernatant was measured using the BCA protein assay. For western blot analysis, 20 μg of proteins were separated by 10% SDS–PAGE at 150 V for 2 h and blotted onto 0.22 mm nitrocellulose membranes at 90 mA for 16 h. The membranes were first blocked for 2 h in TRIS buffered saline solution (TBST, composition: TRIS 10 mM, NaCl 150 mM, 0.1% Tween 20) plus 5% low fat dry milk (TBSTM) and then incubated with the appropriate antibody diluted 1:1000 in TBSTM, for 16 h at 4°C under gentle agitation. The membranes were rinsed three times in TBST and then incubated for 2 h at 21°C with the secondary antibody diluted 1:10000 in TBSTM. Membranes were then rinsed three times in TBST and luminescence was detected by using the appropriate kit, and densitometric analysis was performed as previously reported [18].

### Statistical analysis

Data are shown as means ± standard deviation (SD) unless otherwise indicated. Statistical significance of the differences was assessed by two-tailed Student’s *t* test for paired data or by One-way analysis of variance followed by Dunnett’s Multiple Comparison Test as appropriate. Calculations were performed using a commercial software (GraphPad Prism version 5.00 for Windows, GraphPad Software, San Diego California USA, http://www.graphpad.com).

## Results

### Viability and apoptosis

Viable cells, measured after 24 h, were 81.7 ± 6.0% in basal conditions and 90.2 ± 3.7% in the presence of VEGF, EGF, IGF-I, and FGF2 (n = 4, P = 0.066 *vs* basal conditions). Early apoptotic cells were, respectively, 10.8 ± 2.0% and 6.5 ± 3.4% (n = 4, P = 0.117), late apoptotic/necrotic cells were 5.4 ± 3.5% and 1.7 ± 0.3% (n = 4, P = 0.082) and necrotic cells were 2.5 ± 1.5% and 1.6 ± 0.8% (n = 4, P = 0.430). The presence of cyclo[DKP-RGD] **1** in the 1×10^−12^-1×10^−6^ M did not affect the percentage of viable, early apoptotic, late apoptotic/necrotic or necrotic cells to any significant extent in either experimental conditions (with cyclo[DKP-RGD] **1** 1×10^−6^ M, viable cells: 85.4 ± 3.4% and 86.8 ± 9.2%; early apoptotic cells: 10.5 ± 3.1% and 9.1 ± 7.7%; late apoptotic/necrotic cells: 2.7 ± 0.6% and 2.4 ± 0.9%; necrotic cells: 1.8 ± 0.8% and 1.9 ± 1.1%; in all the cases, n = 4 and P > 0.05 vs control).

### Proliferation

HUVEC proliferation in basal conditions was 0.25 ± 0.18 O.D. and increased up to 1.85 ± 0.50 O.D. in the presence of VEGF, EGF, IGF-I, and FGF2 (n = 3–6, *P* < 0.05). Cell incubation with *cyclo*[DKP-RGD] **1** up to 1×10^−5^ M did not significantly affect either basal or stimulated proliferation (data not shown).

### Migration

**Figure 2:**
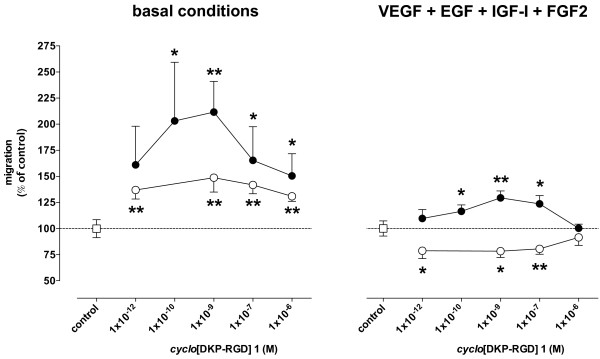
**Effect of *cyclo*[DKP-RGD] 1 on HUVEC migration in the Boyden chamber assay.** Cells were placed in the top compartment. Empty circles: *cyclo*[DKP-RGD] **1** placed in the top compartment. Filled circles: *cyclo*[DKP-RGD] **1** placed in the bottom compartment. Data are means ± SEM of 5–17 separate experiments. * = P < 0.05 and ** = P < 0.01 vs respective control.

Spontaneous migration of HUVEC was 25.2 ± 9.5 μm and increased by 87.8 ± 53.7%, up to 44.9 ± 13.4 μm in the presence of VEGF, EGF, IGF-I, and FGF2 in the bottom chamber (n = 17, *P* < 0.001 *vs* basal conditions). When *cyclo*[DKP-RGD] **1** was added in the top chamber, i.e. together with HUVEC, spontaneous migration was increased and stimulated migration was decreased, while when it was added in the bottom chamber both spontaneous and stimulated migration were increased (Figure 2).

### Angiogenesis

**Figure 3:**
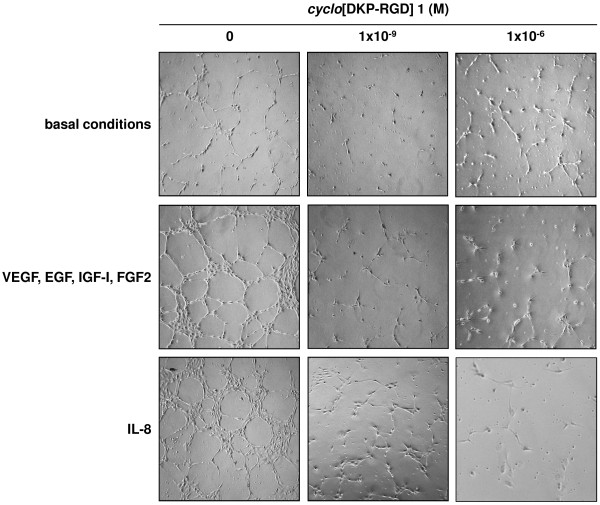
**Representative phase contrast photomicrographs of HUVEC plated on Matrigel in basal conditions or in the presence of VEGF, EGF, IGF-I, and FGF2 or IL-8, without and with *cyclo*** **[DKP-RGD] 1 at different concentrations.**

HUVEC under basal conditions did not show any significant network formation. Addition of VEGF, EGF, IGF-I, and FGF2 induced a significant network formation, which was even higher when cells were treated with IL-8 (Figure 3).

**Figure 4:**
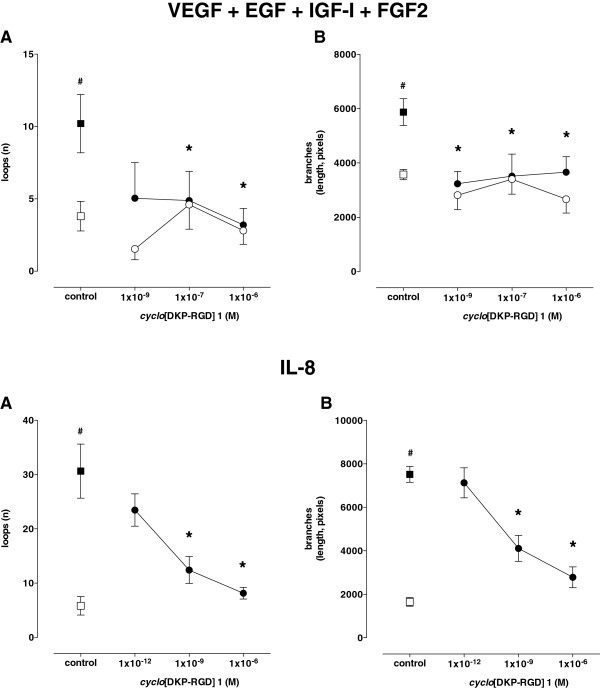
**Effect of *cyclo*[DKP-RGD] 1 on HUVEC angiogenesis induced by VEGF, EGF, IGF-I, and FGF2 (upper panels) or IL-8 (lower panels).** Angiogenesis was evaluated as both number of loops **(A)** and length of branches **(B)**. Empty symbols: basal conditions; filled symbols: stimulated conditions. Data are means ± SEM of 3–5 separate experiments. # = P < 0.01 vs basal conditions, * = P < 0.01 vs respective control.

Coincubation with *cyclo*[DKP-RGD] **1** did not significantly affect angiogenesis of HUVEC under basal conditions (Figure 3b-d), however it significantly and profoundly decreased the effect of VEGF, EGF, IGF-I, and FGF2 (Figure 3 and Figure 4, panel A) as well as the effect of IL-8 (Figure 3 and Figure 4, panel B).

### Expression of mRNA for α_v_, β_3_ and β_5_ integrin subunits

HUVEC expressed comparable amounts of the mRNA for α_v_, β_3_ and β_5_ integrin subunits in both basal conditions and after treatment with VEGF, EGF, IGF-I, and FGF2, and coincubation with 1×10^−6^ M *cyclo*[DKP-RGD] **1** did not affect mRNA expression of any of the subunits in either experimental conditions (Table 2).

**Table 2:** **Real time PCR analysis of the expression of mRNA for the integrin subunits α**_**v**_**, β**_**3 **_**and β**_**5 **_**in HUVEC cultured for 5 h in basal conditions and with VEGF, EGF, IGF, and FGF, alone (control) or in the presence of 1 μM *cyclo*** **[DKP-RGD] 1**

**Subunit**	**control**	**+ *cyclo*** **[DKP-RGD] 1**		
	***2***^***-Δct ***^ ***×10***^***2***^	***2***^***-Δct ***^ ***×10***^***2***^	***Ratio vs Control***	***P vs Control***
**A.** Basal conditions				
α_v_	6.65 ± 6.41	6.59 ± 5.24	1.32 ± 0.57	0.944
β_3_	0.89 ± 0.87	0.91 ± 0.76	1.08 ± 0.27	0.928
β_5_	1.36 ± 1.18	1.52 ± 1.34	1.08 ± 0.07	0.225
**B.** With VEGF, EGF, IGF, and FGF				
α_v_	13.31 ± 12.80	15.09 ± 10.66	1.28 ± 0.42	0.494
β_3_	1.15 ± 1.07	1.41 ± 1.07	1.43 ± 0.35	0.288
β_5_	1.23 ± 1.25	1.31 ± 1.13	1.34 ± 0.46	0.461

Data are means ± SD of 3 separate experiments.

### Akt phosphorylation

**Figure 5:**
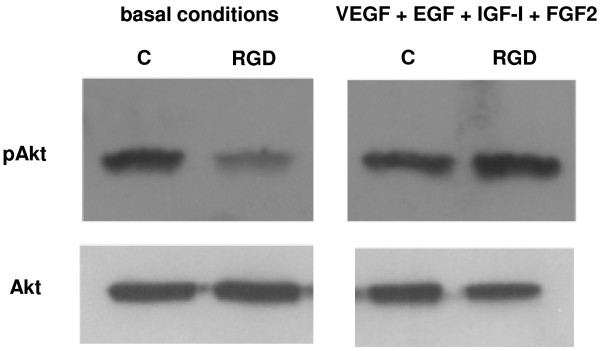
**Western blot analysis of Akt phosphorylation in HUVEC cultured for 5 h in basal conditions and with VEGF, EGF, IGF-I, and FGF2, alone (control, C) or in the presence of 1 μM *cyclo*[DKP-RGD] 1 (RGD).** Data are from one representative of 3 separate experiments.

Treatment of HUVEC with 1×10^−6^ M *cyclo*[DKP-RGD] **1** in basal conditions reduced phosphorylated Akt, from 16241.7 ± 1763.3 to 8702.7 ± 2008.7 optical density arbitrary units, down to 53.2 ± 7.9% of control (n = 3, P = 0.001), without however any significant effect in the presence of VEGF, EGF, IGF-I, and FGF2 (15406.0 ± 1218.8 to 15174.7 ± 663.9 optical density arbitrary units, n = 3, P = 0.735) (Figure 5).

## Discussion

HUVEC represent a valid *in vitro* model which provides seminal insights into the cellular and molecular events leading to neovascularization in response to inflammation and hypoxia in cancer, ischemic events, and in embryogenesis [19]. As anticipated in the introduction, integrins are key actors in angiogenesis and vascular homeostasis, acting as promoters of endothelial cell-matrix interactions [20]. It has been recognized that pharmacological inhibition of the α_v_β_3_ subtype suppresses angiogenesis in many experimental models and α_v_β_3_ antagonists (i.e. antibodies, peptides and peptidomimetics) are being developed as antiangiogenic drugs [21]. It is known that integrins α_v_β_3_ and α_v_β_5_ are expressed on HUVEC [22]; as a consequence these cells represent a suitable model to study the effects of agents acting on such targets. In the present study we used HUVEC to test the ability of the peptidomimetic integrin ligand *cyclo*[DKP-RGD] **1** to affect the key steps of the angiogenic process by evaluating its effects on proliferation, migration and capillary-like network formation. Some of us previously showed that *cyclo*[DKP-RGD] **1** inhibits vitronectin binding to α_v_β_3_ and α_v_β_5_ integrins with IC_50_ of 4.5 ± 1.1 nM and 149 ± 25 nM respectively [16].

In our experiments, the effects of *cyclo*[DKP-RGD] **1** on HUVEC activity were tested in resting conditions as well as in the presence of a culture medium enriched with growth factors known to promote angiogenesis such as VEGF, EGF, IGF-I and FGF2 or after addition of the pro-inflammatory chemokine IL-8, which has a key role in the regulation of pathological angiogenesis [2,3]. According to our results, *cyclo*[DKP-RGD] **1** is indeed able to strongly inhibit angiogenesis, as indicated by the reduction of network formation (*vide infra*), and this occurs without affecting cell viability, apoptosis or proliferation. Most anti-angiogenic compounds acting through the inhibition of integrin function, such as cilengitide, exhibit cytotoxic activity in the same or very close concentration range [23]. In our experimental conditions, our compound did not affect cell viability and apoptosis or cell proliferation, suggesting that its antiangiogenic activity is likely independent from cytotoxicity. This latter observation deserves further consideration because angiogenesis represents a key step in some pathological conditions beyond tumour growth. For example atheromatous plaque vulnerability is closely related to neoangiogenesis [24]; in this latter case a cytotoxic effect exerted by an antiangiogenic compound could represent a risk for adverse effects. On the other hand, the lack of cytotoxic effects by *cyclo*[DKP-RGD] **1** was also observed in several different cell-lines such as ovarian carcinoma IGROV-1 or SKOV3, human pancreatic carcinoma PANC-1 and MIA-PaCa2, human osteosarcoma U2-OS [25], and can be considered therefore as a general feature of our compound. Whether this lack of cytotoxicity might be suggestive of reduced toxicity and increased tolerability *in vivo* in different pathological conditions needs to be assessed in specific studies.

Investigation of the specific mechanisms responsible for the antiangiogenic effects of *cyclo*[DKP-RGD] **1** was beyond the purpose of the present study; nonetheless, according to our results, this compound did not affect the mRNA levels for the integrin subunits α_v_, β_3_ and β_5_, which are the main targets of its action, but it effectively inhibited the phosphorylation of Akt, a serine/threonine-specific protein kinase that plays a key role in the regulation of vascular homeostasis and angiogenesis [17]. The fact that the inhibition of Akt phosphorylation is only detected under basal conditions may be explained considering that, in the presence of growth factors converging on the same intracellular signalling pathway, the inhibitory effect exerted by *cyclo*[DKP-RGD] **1** is probably overcome. Inhibition of Akt phosphorylation by *cyclo*[DKP-RGD] **1** is likely the results of disruption of proper endothelial cell–extracellular matrix attachment, due to integrin engagement by *cyclo*[DKP-RGD] **1**. Indeed, it has already been reported that antagonists against α_v_β_3_ or α_v_β_5_ integrin interfere with angiogenesis induced by several growth factors: for instance, α_v_β_3_ integrin associates with VEGF and platelet-derived growth factor (PDGF) receptors and potentiates VEGF or PDGF signaling, respectively [26].

Disruption of integrin functions may possibly explain also the effects of *cyclo*[DKP-RGD] **1** on HUVEC migration. Indeed, in the presence of *cyclo*[DKP-RGD] **1** migration was increased in resting conditions but it was decreased in stimulated conditions when the compound was added in the top compartment of the Boyden chamber, together with the cells, while it was increased in both resting and stimulated conditions when the compound was added in the bottom compartment. As a temptative explanation, we propose that increased migration results from the direct inhibitory effect of *cyclo*[DKP-RGD] **1** on integrins α_v_β_3_ and α_v_β_5_, resulting in reduced cell anchorage to surfaces. On the other side, the slight decrease of stimulated migration and the reduced increase of spontaneous migration when *cyclo*[DKP-RGD] **1** was added in the same compartment in which the cells were placed might imply also a slight chemoattractant effect of this compound, which would therefore not only increase cell random migration through decreased integrin-mediated attachment to the surfaces, but also attract the cells along its concentration gradient. The *in vivo* relevance of such effect, where no concentration gradient is expected to occur, is however questionable. Remarkably, the effect exerted by *cyclo*[DKP-RGD] **1** was apparently bell-shaped, with a peak at about 1×10^−9^ M (which however was not observed in the angiogenesis assay). Whether this finding implies different modes of action depending on the extension of integrin engagement on the cell surface, it should be established in specific experiments. Disruption of integrin function could therefore explain both the increased migration and the anti-angiogenic activity exerted by *cyclo*[DKP-RGD] **1**. A similar effect was observed by Mrksich and co-workers [27], who promoted cell migration on self-assembled monolayers containing immobilized cyclic RGD by addition of exogenous linear RGD ligands [27].

In our experiments, *cyclo*[DKP-RGD] **1** effectively inhibited angiogenesis induced by the growth factors VEGF, EGF, IGF-I and FGF2, as well as by IL-8. All these proangiogenic agents act through distinct membrane receptors [28,29] which result in the activation of extensively overlapping intracellular cascades finally activating common effector molecules, such as NF-κB or HIF-1 [28]. In addition, recent evidences indicate that direct interactions may occur between integrin activated pathways and signalling from VEGF receptors [30] and EGF receptors [31]. Collectively, in the light of such observations, our results support the ability of *cyclo*[DKP-RGD] **1** to block common mechanisms, resulting in the effective inhibition of angiogenesis triggered by multiple agents. Angiogenesis is a process that occurs not only in cancer, but also in many other critical diseases such as atherosclerosis [32], and the relevance of *cyclo*[DKP-RGD] **1**-induced effects in such conditions needs careful assessment.

In conclusion, the data of the present study show that the novel compound *cyclo*[DKP-RGD] **1**, an α_v_β_3_ and α_v_β_5_ integrin ligand, effectively inhibits angiogenic processes in HUVEC, possibly through mechanisms involving reduced Akt phosphorylation and disruption of integrin-mediated adhesion, without affecting their viability and proliferation. We propose therefore this compound as a candidate modulator of angiogenesis occurring in different conditions, possibly devoid of the adverse effects of cytotoxic analogues. Further studies clarifying the *in vivo* activity of *cyclo*[DKP-RGD] **1**, including a complete toxicological assessment, as well as a thorough investigation of the intracellular pathways involved its effects are currently underway in order to evaluate its possible potential applications as a novel pharmacotherapeutic compound.

## Competing interests

The authors declare that they have no competing interests.

## Authors’ contributions

RF and LS = study design, performing all in vitro experiments and data handling. UP = Study design and manuscript preparation^.^ MP = in vitro experiments on morphogenesis and data handling. ER = flow cytometry analysis and data handling^.^ MP and MCG = real time PCR and Western Blot experiments and data handling^.^ MC = Study design, data handling, manuscript preparation and revision^.^ FM = Study design, data handling, manuscript preparation and revision.
